# Selective eye fixations on diagnostic face regions of dynamic emotional expressions: KDEF-dyn database

**DOI:** 10.1038/s41598-018-35259-w

**Published:** 2018-11-19

**Authors:** Manuel G. Calvo, Andrés Fernández-Martín, Aida Gutiérrez-García, Daniel Lundqvist

**Affiliations:** 1 Department of Cognitive Psychology, Universidad de La Laguna, Tenerife, Spain; 2 Instituto Universitario de Neurociencia (IUNE), Universidad de La Laguna, Tenerife, Spain; 3 Department of Health Sciences, Universidad Internacional de La Rioja, Logroño, Spain; 4 Department of Health Sciences, Universidad de Burgos, Burgos, Spain; 5 Karolinska Institutet, Stockholm, Sweden

## Abstract

Prior research using static facial stimuli (photographs) has identified diagnostic face regions (i.e., functional for recognition) of emotional expressions. In the current study, we aimed to determine attentional orienting, engagement, and time course of fixation on diagnostic regions. To this end, we assessed the eye movements of observers inspecting dynamic expressions that changed from a neutral to an emotional face. A new stimulus set (KDEF-dyn) was developed, which comprises 240 video-clips of 40 human models portraying six basic emotions (happy, sad, angry, fearful, disgusted, and surprised). For validation purposes, 72 observers categorized the expressions while gaze behavior was measured (probability of first fixation, entry time, gaze duration, and number of fixations). Specific visual scanpath profiles characterized each emotional expression: The eye region was looked at earlier and longer for angry and sad faces; the mouth region, for happy faces; and the nose/cheek region, for disgusted faces; the eye and the mouth regions attracted attention in a more balanced manner for surprise and fear. These profiles reflected enhanced selective attention to expression-specific diagnostic face regions. The KDEF-dyn stimuli and the validation data will be available to the scientific community as a useful tool for research on emotional facial expression processing.

## Introduction

Facial expressions are assumed to convey information about a person’s current feelings and motives, intentions and action tendencies. Most research on expression recognition has been conducted under a categorical view, using six basic expressions: happiness, anger, sadness, fear, disgust and surprise^1^ (for a review, see^2^). Emotion recognition relies on expression-specific diagnostic (i.e., distinctive) features, in that they are necessary or sufficient for recognition of the respective emotion: Anger and sadness are more recognizable from the eye region (e.g., frowning), whereas happiness and disgust are more recognizable from the mouth region (e.g., smiling), while recognition of fear and surprise depends on both regions^3–8^. In the current study, we aimed to determine the profile of overt attentional orienting to and engagement with such expression-diagnostic features; that is, *whether*, *when*, and *how long* they selectively attract eye fixations from observers. Importantly, we addressed this issue for *dynamic* facial expressions, thus extending typical approaches using photographic stimuli.

Prior eyetracking research using photographs of *static* expressions has provided non-conclusive evidence regarding the pattern and role of selective visual attention to facial features. First, during expression recognition, gaze allocation is often biased towards diagnostic face regions (e.g., the eye region receives more attention in sad and angry faces, whereas the mouth region receives more attention in happy and disgusted faces^3,8–11^). However, in other studies, the proportion of fixation on the different face areas was modulated by expression less consistently or was not affected^12–15^. Second, increased visual attention to diagnostic facial features is correlated with improved recognition performance^16^. Looking at the mouth region contributes to recognition of happiness^3,8^ and disgust^8^, and looking at the eye/brow area contributes to recognition of sadness^3^ and anger^8^. However, results are less consistent for other emotions, and the role of fixation on diagnostic regions depends on expressive intensity, with recognition of subtle emotions being facilitated by fixations on the eyes (and a lesser contribution by the mouth), whereas recognition of extreme emotions is less dependent on fixations^14^.

Nonetheless, facial expressions are generally *dynamic* in daily social interaction. In addition, research has shown that motion benefits facial affect recognition (see^17–19^). Consistently, relative to static expressions, the viewing of dynamic expressions enhances brain activity in regions associated with processing of social-relevant (superior temporal sulci) and emotion-relevant (amygdala) information^20,21^, which might explain the dynamic expression recognition advantage. Accordingly, it is important to investigate oculomotor behavior during the recognition of this type of expressions. To our knowledge, only a few studies have measured fixation patterns during dynamic facial expression processing, with non-convergent results. Lischke *et al*.^22^ reported an enhanced gaze duration bias towards the eye region of angry, sad, and fearful faces, while gaze duration was longer for the mouth region of happy faces (although differences were not statistically analyzed). In contrast, in the Blais, Fiset, Roy, Saumure-Régimbald, and Gosselin^23^ study, fixation patterns did not differ across six basic expressions and were not linked to a differential use of facial features during recognition.

It is, however, possible that the lack of fixation differences across expressions in the Blais *et al*.^23^ study was due to the use of (a) a short stimulus display (500 ms), thereby limiting the number of fixations (two fixations per trial); and (b) a small stimulus size (width: 5.72°), as the eyes and mouth were close to (1.7° and 2.1°) the center of the face (initial fixation location), and thus they could be seen in parafoveal vision (which then probably curtailed saccades). If so, such stimulus conditions might have reduced sensitivity of measurement. Yet, it must be noted that—in the absence of differences as a function of expression—fixations did vary as a function of display mode, with more fixations on the left eye and the mouth in the static than in the dynamic condition^23^. To clarify this issue, first, we used longer stimulus displays (1,033 ms), thus approximating the typical duration of expression unfolding for most basic emotions^19,24^. Second, we used larger face stimuli (8.8° width × 11.6° height, at an 80-cm viewing distance), which approximates the size of a real face (i.e., 13.8 × 18.5 cm, viewed from 1 m). In fact, in the Lischke *et al*.^22^ study (where fixation differences did occur as a function of expression), the stimulus display was longer (800 ms) and the size was larger (17° × 23.6°) than in the Blais *et al*.^23^ study.

An additional contribution of the current study involves the recollection of norming eyetracking data for each of 240 video-clip stimuli that will be available as a new dynamic expression stimulus set (KDEF-dyn) for other researchers. A number of dynamic expression databases have been developed (for a review, see^25^). To our knowledge, however, for none of them have eyetracking measures been obtained. Thus we make a contribution by devising a facial expression database for which eye movements and fixations are assessed while observers scan faces during emotional expression categorization. The current approach will provide information about the time course of selective attention to face regions, in terms of both orienting (as measured by the probabilities of entry and of first fixation on each region) and engagement (as indicated by gaze duration and number of fixations). If observers move their eyes to face regions that maximize performance determining the emotional state of a face^26^, then regions with expression-specific diagnostic features should receive selective attention, in the form of *earlier* orienting or *longer* engagement, relative to other regions. Thus, in a confirmatory approach, we predict enhanced attention to the eye region of angry and sad faces, to the mouth region of happy and disgusted faces, and a more balanced attention to the eyes and mouth of fearful and surprised faces. In an exploratory approach, we aim to examine how each attentional component, i.e., orienting and engagement, is affected.

We used a dynamic version (KDEF-dyn) of the original (static) Karolinska Directed Emotional Faces (KDEF) database^27^. The photographic KDEF stimuli have been examined in large norming studies^28,29^, and widely employed in behavioral^30–32^ and neurophysiological^33–35^ research (according to Google Scholar, the KDEF has been cited in over 2,000 publications). We built *dynamic* expressions by applying morphing animation to the KDEF photographs, whereby a neutral face changed towards a full-blown emotional face, trying to mimic real-life expressions and the average natural speed of emotional expression unfolding^24,36^. This approach provides fine-grained control and standardization of duration, speed, and intensity. Further, dynamically morphed facial expression stimuli have often been employed in behavioral^18,24,37,38^ and neurophysiological^39–42^ research. Although this type of expressions may not convey the same naturalness as online video recordings, some studies indicate that natural expressions unfold in a uniform and ballistic way^43,44^, thus actually sharing properties with morphed dynamic expressions.

## Method

### Participants

Seventy-two university undergraduates (40 female; 32 male; aged 18 to 30 years: *M* = 21.3) from different courses participated for course credit or payment, after providing written informed consent. A power calculation using G*Power (version 3.1.9.2^45^) showed that 42 participants would be sufficient to detect a medium effect size (Cohen’s *d* = 0.60) at α = 0.05, with power of 0.98, in an a priori analysis of repeated measures within factors (type of expression and face region) ANOVA. As this was a norming study of stimulus materials, a larger participant sample (i.e., 72) was used to obtain stable and representative mean scores. The study was approved by the University of La Laguna ethics committee (CEIBA, protocol number 2017–0227), and conducted in accordance with the WMA Declaration of Helsinki 2008.

### Stimuli

The color photographs of 40 people (20 female; 20 male) from the KDEF set^27^, each displaying six basic expressions (happiness, sadness, anger, fear, disgust, and surprise), were used (see the KDEF identities in Supplemental Datasets S1A and S1B). For the current study, 240 dynamic video-clip versions (1,033 ms duration) of the original photographs were constructed. The face stimuli were subjected to morphing by means of FantaMorph© software (v. 5.4.2, Abrosoft, Beijing, China). For each expression and poser, we created a sequence of 31 (33.33-ms) frames, with intensity increasing at a rate of 30 frames per second, starting with a neutral face as the first frame (frame 0; original KDEF), and ending with the peak of an emotional face (either happy, sad, etc.) in the last frame (frame 30; original KDEF). A similar procedure and display duration has been used in prior research^19,46,47^. The stimuli and the norming data are available at http://kdef.se/versions.html; KDEF-dyn II).

### Procedure

All 72 participants were presented with all 240 video-clips (40 posers × 6 expressions) in six blocks of 40 trials each. Block order was counterbalanced, and trial order and type of expression were randomized for each participant. The stimuli were displayed on a computer screen by means of SMI Experiment Center™ 3.6 software (SensoMotoric Instruments GmbH, Teltow, Germany). Participants were asked to indicate which of six basic expressions was shown on each trial by pressing a key out of six. Twelve video-clips served as practice trials, with two new models showing each expression.

The sequence of events on each trial was as follows. After an initial 500-ms central fixation cross on a screen, a video-clip showed a facial expression unfolding for 1,033 ms. The face subtended a visual angle of 11.6° (height) × 8.8° (width) at a 80-cm viewing distance. Following face offset, six small boxes appeared horizontally on the screen for responding, with each box associated to a number/label (e.g., 4: happy; 5: sad, etc.). For expression categorization, participants pressed one key (from 4 to 9) in the upper row of a standard computer keyboard with their dominant index finger. The assignment of expressions to keys was counterbalanced. The chosen response and reaction times (from the offset of the video-clip) were recorded. There was a 1,500-ms intertrial interval.

### Design and measures

A within-subjects experimental design was used, with expression (happiness, sadness, anger, fear, disgust, and surprise) as a factor. As dependent variables, we measured three aspects of expression categorization performance: (a) hits, i.e., the probability that responses coincided with the displayed expression (e.g., responding “happy” when the face stimulus was intended to convey happiness); (b) reaction times (RTs) for hits; and (c) type of confusions, i.e., the probability that each target stimulus (the displayed expression) was categorized as each of the other five, non-target expressions (e.g., if the target was anger in a trial, the five non-targets were happiness, sadness, disgust, fear, and surprise).

Eye-movements were recorded by means of a 500-Hz (binocular; spatial resolution: 0.03°; gaze position accuracy: 0.4°) RED system eyetracker (SensoMotoric Instruments, SMI, Teltow, Germany). The following measures were obtained: (a) probability that the *first* fixation on the face (following the initial fixation on the central fixation point on the nose) landed on each of three regions of interest (see below); (b) probability of *entry* in each region during the display period (entry *times* are also reported in Supplemental Datasets S1A), but were not analyzed because some regions were not looked at by all viewers; thus the mean entry *times* are informative only by taking the *probability* of entry into account); (c) number of *fixations* (if ≥80 ms duration) on each region; and (d) gaze *duration* or total fixation time on each region. The probability of first fixation and entry assessed attentional *orienting*. The number of fixations and gaze duration assessed attentional *engagement*. In addition, to examine the *time course* of selective attention to face regions along expression unfolding, we computed the proportion of gaze duration for each face region during each of 10 consecutive intervals of 100 ms each (i.e., from 1 to 100 ms, from 101 to 200 ms, etc.) across the 1,033-ms display (the final 33 ms were not included). *Net* gaze duration was obtained and analyzed after saccades and blinks were excluded. For saccade and fixation detection parameters, we used a velocity-based algorithm with a 40°/s peak velocity threshold and 80 ms for minimum fixation duration (for details, see^48^).

**Figure 1:**
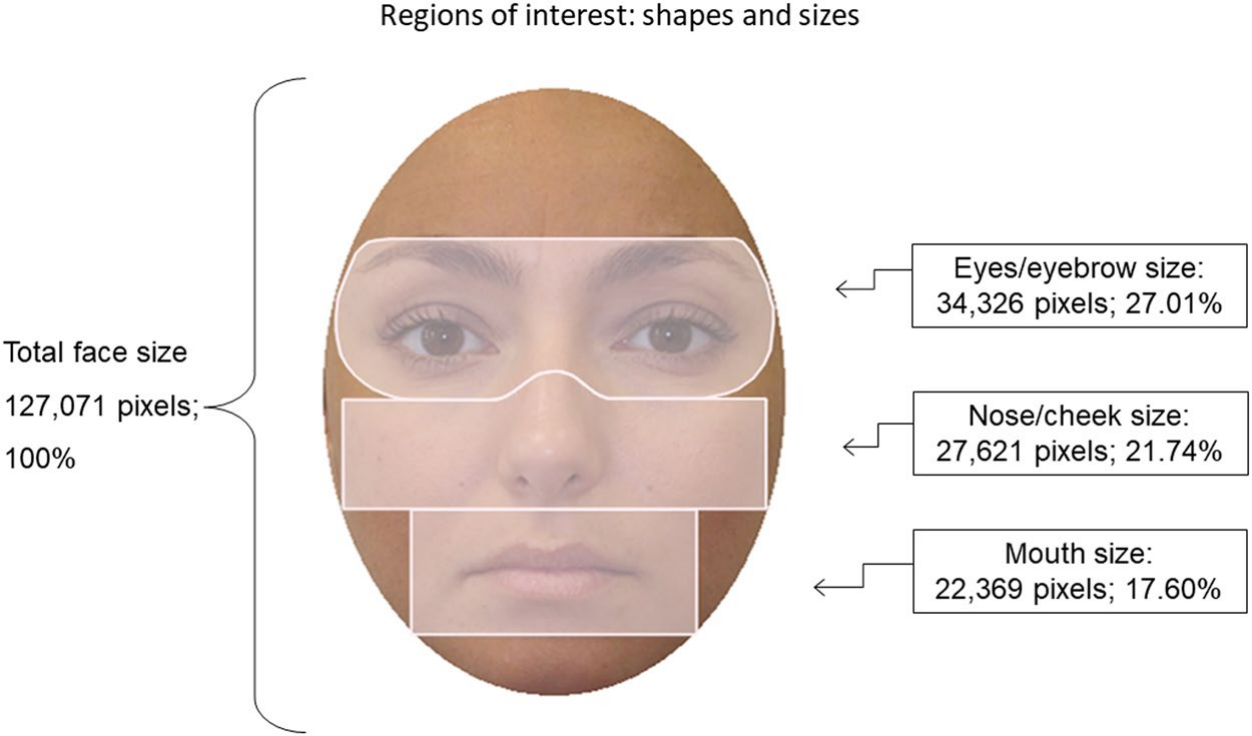
Regions of interest (with shapes and sizes, in pixels) of face stimuli used for eye-fixation assessment.

Three face regions of interest were defined: eye and eyebrow (henceforth, eye region), nose/cheek (henceforth, nose), and mouth (see their sizes and shapes in Fig. 1). About 97% of total fixations occurred within these three regions (the forehead and the chin were excluded because they received only 1.2% of fixations).

## Results

Given that one major aim of the study was to obtain and provide other researchers with validation measures for *each stimulus* in the KDEF-dyn database, the statistical analyses were performed by items, with the 240 video-clip stimuli as the units of analysis (and scores averaged for the 72 participants). For all the following analyses, the post hoc multiple comparisons across expressions used a familywise error rate (FWER) procedure, with single step (i.e., equivalent adjustments made to each *p* value) Bonferroni corrections (with a *p* < 0.05 threshold).

### Analyses of expression recognition performance and confusions

For the probability of *accurate* responses, a one-way (6: Expression stimulus: happiness, surprise, anger, sadness, disgust, and fear) ANOVA yielded significant effects, *F*(5, 234) = 39.34, *p* < 0.001, η_p_^2^ = 0.46. Post hoc contrasts revealed better recognition of happiness, surprise, and anger (which did not differ from one another), relative to sadness and disgust (which did not differ), which were recognized better than fear (see Table 1, Hits row). The correct response *reaction times*, *F*(5, 234) = 50.26, *p* < 0.001, η_p_^2^ = 0.52, were faster for happiness than for all the other expressions, followed by surprise, followed by disgust, anger, and sadness (which did not differ from one another), and fear was recognized most slowly (see Table 1, Hit RTs row).

**Table 1:** Mean Proportion (%; and *SD*s in parenthesis) of Responses (Hits and Confusions, and Hit Reaction Times) for each Target (Stimulus) Expression.

Stimulus Expression	Response Expression					
	Happiness	Surprise	Anger	Sadness	Disgust	Fear
Happiness	**97.6** ^**a**^ **(4.9)**	1.5^b^ (4.8)	0.1^b^ (0.4)	0.0^b^ (0.3)	0.8^b^ (1)	0.0^b^ (0.2)
Surprise	1.7^bc^ (2.1)	**95.3** ^**a**^ **(4.5)**	0.2^cd^ (0.6)	0.1^d^ (0.5)	0.4^cd^ (0.8)	2.3^b^ (3.6)
Anger	0.0^c^ (0.3)	0.6^c^ (1.6)	**92.7** ^**a**^ **(8.8)**	1.2^bc^ (2.1)	3.8^b^ (6)	1.7^b^ (1.8)
Sadness	0.3^d^ (1.2)	0.1^d^ (2.2)	1.4^d^ (2.6)	**75.2** ^**a**^ **(20.5)**	7.7^c^ (10.9)	14.3^b^ (12,4)
Disgust	0.1^d^ (0.4)	0.8^cd^ (1.4)	10.8^b^ (15.9)	4.9^bc^ (10.1)	**80.4** ^**a**^ **(18.4)**	3.0^b^ (5.8)
Fear	0.5^e^ (0.8)	26.0^b^ (23.7)	0.8^de^ (1.6)	2.4^d^ (3.5)	10.5^c^ (12.4)	**59.8** ^**a**^ **(20.4)**
**Hits**	97.6^a^ (4.9)	95.3^a^ (4.5)	92.7^a^ (8.8)	75.2^b^ (20.5)	80.4^b^ (18.4)	59.8^c^ (20.4)
**Hit RTs**	823^a^ (80)	958^b^ (116)	1,154^c^ (148)	1,239^c^ (210)	1,149^c^ (235)	1,429^d^ (273)

*Note*. For each expression stimulus category, scores with different letters (on the same row) are significantly different in post hoc multiple contrasts (*p* < 0.05, Bonferroni corrected); expressions sharing a letter are equivalent. Boldface for hits.

A 6 (Expression stimulus) × 6 (Expression response) ANOVA on *confusions* yielded interactive effects, *F*(25, 1170) = 581.13, *p* < 0.001, η_p_^2^ = 0.92, which were decomposed by one-way (6: Expression response) ANOVAs for each expression stimulus separately (see Table 1). Facial *happiness*, *F*(5, 195) = 6489.44, *p* < 0.001, η_p_^2^ = 0.99, was minimally confused. *Surprise*, *F*(5, 195) = 6781.17, *p* < 0.001, η_p_^2^ = 0.99, was slightly confused with fear and happiness; *anger*, *F*(5, 195) = 2231.79, *p* < 0.001, η_p_^2^ = 0.98, with disgust and fear; *sadness*, *F*(5, 195) = 241.36, *p* < 0.001, η_p_^2^ = 0.86, with fear and disgust; *disgust*, *F*(5, 195) = 289.88, *p* < 0.001, η_p_^2^ = 0.87, with anger, sadness, and fear; and *fear*, *F*(5, 195) = 94.24, *p* < 0.001, η_p_^2^ = 0.71, was confused mainly with surprise.

### Analyses of eye movement measures

A 6 (Expression stimulus) ×3 (Face region: eyes, nose/cheek, and mouth) ANOVA was conducted on each eye-movement measure. The significant interactions were decomposed by means of one-way (6: Expression) ANOVAs for each region. Post hoc multiple comparisons examined how much the processing of each expression relied on a face region more than other expressions did. The critical comparisons involved contrasts across expressions for each region (which was of identical size for all the expressions), rather than across regions for each expression (as regions were different in size, thus probably affecting gaze behavior). The first fixation on the nose was removed as uninformative, given that the initial fixation point was located on this region.

For *probability of first fixation*, effects of region, *F*(2, 468) = 2361.70, *p* < 0.001, η_p_^2^ = 0.91, but not of expression, *F*(5, 234) = 1.90, *p* = 0.095, *ns*, and an interaction, *F*(10, 468) = 9.75, *p* < 0.001, η_p_^2^ = 0.17, emerged. The one-way (Expression) ANOVA yielded effects for the eye region, *F*(5, 234) = 10.26, *p* < 0.001, η_p_^2^ = 0.18, and the mouth, *F*(5, 234) = 17.05, *p* < 0.001, η_p_^2^ = 0.27, but not the nose, *F*(5, 234) = 1.56, *p* = 0.17, *ns*. As indicated in Table 2 (means and multiple contrasts), (a) the *eye* region was more likely to be fixated first in angry faces relative all the others, except for sad faces, which, along with surprised, disgusted, and fearful faces, were more likely to be fixated first on the eyes than happy faces were; and (b) the *mouth* region of happy faces was more likely to be fixated first, relative to the other expressions.

**Table 2:** Mean Probability of First Fixation (and *SDs*) on each Face Region for each Expression.

Stimulus Expression	Face Region					
	Eyes		Nose/Cheek		Mouth	
	*M*	*SD*	*M*	*SD*	*M*	*SD*
Happiness	0.478^c^	0.038	0.231^a^	0.045	**0.236** ^**a**^	0.050
Surprise	0.533^b^	0.047	0.218^a^	0.045	0.189^b^	0.048
Anger	**0**.**548**^a^	0.055	0.229^a^	0.050	0.154^c^	0.042
Sadness	**0.542** ^ab^	0.056	0.240^a^	0.046	0.156^c^	0.037
Disgust	0.516^b^	0.050	0.245^a^	0.051	0.168^bc^	0.046
Fear	0.512^b^	0.053	0.230^a^	0.052	0.193^b^	0.055

*Note*. Fixations following the initial fixation on the central fixation point. *Within* each face *region* (on the same column), *across expressions*, scores with different letters are significantly different (*p* < 0.05, Bonferroni corrected); scores sharing a letter are equivalent. Boldface indicates that the region was most characteristic of the respective expression.

For *probability of entries*, effects of region, *F*(2, 468) = 3274.54, *p* < 0.001, η_p_^2^ = 0.93, expression, *F*(5, 234) = 4.66, *p* < 0.001, η_p_^2^ = 0.09, and an interaction, *F*(10, 468) = 47.91, *p* < 0.001, η_p_^2^ = 0.51, emerged. The one-way (Expression) ANOVA yielded effects for the eye region, *F*(5, 234) = 46.04, *p* < 0.001, η_p_^2^ = 0.50, the nose, *F*(5, 234) = 20.03, *p* < 0.001, η_p_^2^ = 0.30, and the mouth, *F*(5, 234) = 37.01, *p* < 0.001, η_p_^2^ = 0.44. As indicated in Table 3 (means and multiple contrasts), (a) the probability of entry in the *eye* region was higher for the angry, sad, and surprised faces than for disgusted and happy faces; (b) it was higher in the *nose* region for happy and disgusted faces than for the others; and (c) it was highest in the *mouth* region for happy faces.

**Table 3:** Mean Probability of Entry (and *SDs*) on each Face Region for each Expression.

Stimulus Expression	Face Region					
	Eyes		Nose/Cheek		Mouth	
	*M*	*SD*	*M*	*SD*	*M*	*SD*
Happiness	0.714^c^	0.076	**0**.**833**^a^	0.041	**0.505** ^**a**^	0.058
Surprise	**0.917** ^**a**^	0.068	0.784^b^	0.045	0.374^b^	0.066
Anger	**0.929** ^**a**^	0.076	0.764^b^	0.045	0.301^c^	0.076
Sadness	**0.923** ^**a**^	0.079	0.759^b^	0.052	0.309^c^	0.084
Disgust	0.846^b^	0.077	**0.828** ^a^	0.036	0.356^b^	0.082
Fear	0.891^ab^	0.083	0.785^b^	0.047	0.358^b^	0.088

*Note. Within* each face *region* (on the same column), *across expressions*, scores with different letters are significantly different (*p* < 0.05, Bonferroni corrected); scores sharing a letter are equivalent. Boldface indicates that the region was most characteristic of the respective expression.

For *gaze duration*, effects of region, *F*(2, 468) = 2007.02, *p* < 0.001, η_p_^2^ = 0.90, but not of expression (*F* < 1), and an interaction, *F*(10, 468) = 42.45, *p* < 0.001, η_p_^2^ = 0.48, emerged. The one-way (Expression) ANOVA yielded effects for the eye region, *F*(5, 234) = 51.76, *p* < 0.001, η_p_^2^ = 0.52, the nose, *F*(5, 234) = 10.60, *p* < 0.001, η_p_^2^ = 0.19, and the mouth, *F*(5, 234) = 49.31, *p* < 0.001, η_p_^2^ = 0.51. As indicated in Table 4 (means and multiple contrasts), (a) the *eye* region was fixated longer in angry and sad faces, relative to the others; (b) the *nose* region, in disgusted faces; and (c) the *mouth*, in happy faces.

**Table 4:** Mean Gaze Duration (and *SDs*; in ms) on each Face Region for each Expression.

Stimulus Expression	Face Region					
	Eyes		Nose/Cheek		Mouth	
	*M*	*SD*	*M*	*SD*	*M*	*SD*
Happiness	286^d^	30	385^ab^	30	**220** ^a^	35
Surprise	390^b^	47	356^c^	37	145^b^	34
Anger	**427** ^a^	53	359^c^	35	113^c^	35
Sadness	**421** ^a^	44	359^c^	39	115^c^	31
Disgust	359^c^	41	**399** ^a^	24	136^b^	36
Fear	383^bc^	51	369^bc^	34	142^b^	39

*Note. Within* each face *region* (on the same column), *across expressions*, scores with different letters are significantly different (*p* < 0.05, Bonferroni corrected); scores sharing a letter are equivalent. Boldface indicates that the region was most characteristic of the respective expression.

For *number of fixations*, effects of region, *F*(2, 468) = 1624.24, *p* < 0.001, η_p_^2^ = 0.87, but not of expression, *F*(5, 234) = 2.06, *p* = 0.071, *ns*, and an interaction, *F*(15, 702) = 39.71, *p* < 0.001, η_p_^2^ = 0.46, appeared. The one-way (Expression) ANOVA yielded effects for the eye region, *F*(5, 234) = 46.11, *p* < 0.001, η_p_^2^ = 0.50, the nose, *F*(5, 234) = 6.87, *p* < 0.001, η_p_^2^ = 0.13, and the mouth, *F*(5, 234) = 36.63, *p* < 0.001, η_p_^2^ = 0.45. As indicated in Table 5 (means and multiple contrasts), (a) the *eye* region was fixated more frequently in angry, sad, and surprised faces; (b) the *nose*, in disgusted and happy faces; and (c) the *mouth*, in happy faces.

**Table 5:** Mean Number of Fixations (and *SDs*) on each Face Region for each Expression.

Stimulus Expression	Face Region					
	Eyes		Nose/Cheek		Mouth	
	*M*	*SD*	*M*	*SD*	*M*	*SD*
Happiness	1.13^d^	0.14	**1.52** ^a^_**1**_	0.13	**0.95** ^a^	0.15
Surprise	1.58^ab^	0.18	1.43^b^	0.16	0.66^b^	0.14
Anger	**1.66** ^a^	0.21	1.41^b^	0.13	0.52^c^	0.15
Sadness	**1.62** ^ab^	0.20	1.40^b^	0.13	0.53^c^	0.17
Disgust	1.43^c^	0.15	**1.53** ^a^	0.11	0.63^b^	0.17
Fear	1.54^bc^	0.20	1.45^ab^	0.13	0.63^b^	0.17

*Note. Within* each face *region* (on the same column), *across expressions*, scores with different letters are significantly different (*p* < 0.05, Bonferroni corrected); scores sharing a letter are equivalent. Boldface indicates that the region was most characteristic of the respective expression.

### Time course of selective attention to expression-diagnostic features

**Figure 2:**
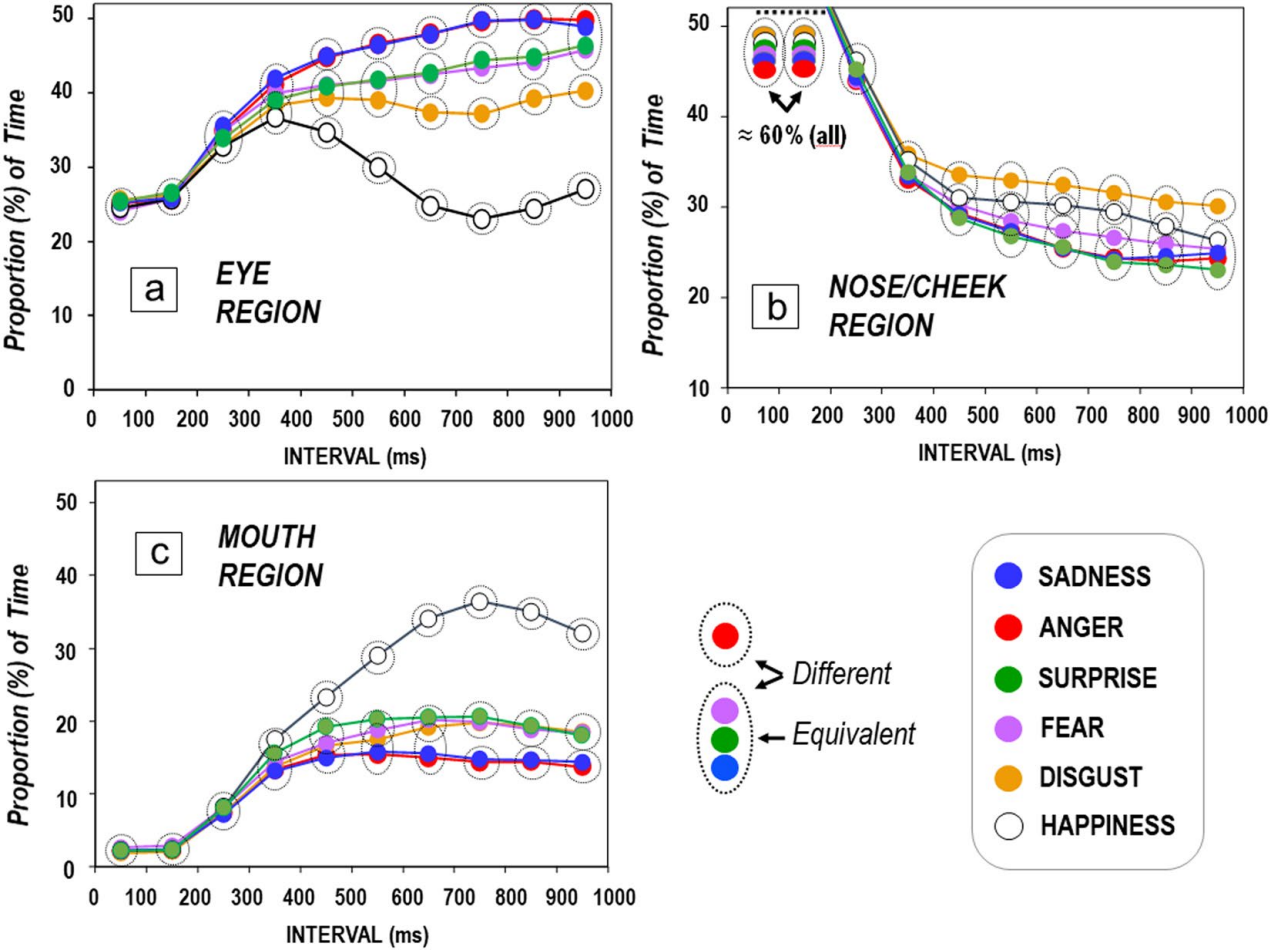
(**a,b,c**) Time course of fixation on each region. Proportion of fixation time on each region (a: Eyes; b: Nose/cheek; c: Mouth) for each facial expression across 10 consecutive 100-ms intervals. For each interval, expressions within a different dotted circle/oval are significantly different from one another (in post hoc multiple contrasts; after *p* < 0.05 Bonferroni corrections); expressions within the same circle/oval are equivalent. For (**b**), the scale has been slightly stretched, to better notice differences between expressions.

An overall ANOVA of Expression (6) by Region (3) by Interval (10) was performed on the proportion of gaze duration for each region during each of 10 consecutive 100-ms intervals across expression unfolding. Effects of region, *F*(2, 702) = 2818.37, *p* < 0.001, η_p_^2^ = 0.89, and interval, *F*(9, 6818) = 8.79, *p* < 0.001, η_p_^2^ = 0.01, were qualified by interactions of region by expression, *F*(10, 702) = 61.10, *p* < 0.001, η_p_^2^ = 0.47, interval by region, *F*(18, 6318) = 2178.64, *p* < 0.001, η_p_^2^ = 0.86, and a three-way interaction, *F*(90, 6318) = 39.62, *p* < 0.001, η_p_^2^ = 0.36 (see Fig. 2a,b,c; see also Supplemental Datasets S1C Tables). To decompose the three-way interaction, two-way ANOVAs of Expression by Interval were run for each region, further followed by one-way ANOVAs testing the effect of Expression in each time window, with post hoc multiple comparisons (*p* < 0.05, Bonferroni corrected). This approach served to determine two aspects of the attentional time course: the *threshold* (i.e., the earliest interval) and the *amplitude* (i.e., for how many intervals) each face region was looked at more for an expression than for the others.

For the *eye region*, effects of expression, *F*(5, 234) = 51.76, *p* < 0.001, η_p_^2^ = 0.53, and interval, *F*(9, 2106) = 556.98, *p* < 0.001, η_p_^2^ = 0.70, and an interaction, *F*(45, 2106) = 35.06, *p* < 0.001, η_p_^2^ = 0.43, appeared. Expression effects were significant for all the intervals from the 301-to-400 ms time window onwards, with statistical significance ranging between *F*(5, 234) = 6.86, *p* < 0.001, η_p_^2^ = 0.13 and *F*(5, 234) = 72.25, *p* < 0.001, η_p_^2^ = 0.61. The post hoc contrasts and the significant differences across expressions within each interval are shown in Fig. 2a. An advantage emerged for *sad* and *angry* expressions, with the *threshold* located at the 401-to-500-ms interval, where their eye regions attracted more fixation time than for all the other expressions, and the *amplitude* of this advantage remained until 900 ms post-stimulus onset. Secondary advantages appeared for surprised and fearful faces, relative to disgusted and happy faces (see Fig. 2a).

For the *nose/cheek region*, effects of expression, *F*(5, 234) = 10.86, *p* < 0.001, η_p_^2^ = 0.19, and interval, *F*(9, 2106) = 2910.16, *p* < 0.001, η_p_^2^ = 0.93, were qualified by an interaction, *F*(45, 2106) = 4.58, *p* < 0.001, η_p_^2^ = 0.09. Expression effects were significant for all the intervals from the 401-to-500 ms time window onwards, ranging between *F*(5, 234) = 5.67, *p* < 0.001, η_p_^2^ = 0.11 and *F*(5, 234) = 15.62, *p* < 0.001, η_p_^2^ = 0.25. The post hoc contrasts and the significant differences across expressions within each interval are shown in Fig. 2b. An advantage emerged for *disgusted* expressions over all the others, except for happy faces, with the *threshold* located at the 401-to-500-ms interval: The mouth/cheek region attracted more fixation time for disgusted faces than for all the other expressions (except happy faces), and the *amplitude* of this advantage remained until the end of the 1,000-ms display.

For the *mouth region*, effects of expression, *F*(5, 234) = 49.96, *p* < 0.001, η_p_^2^ = 0.52, interval, *F*(9, 2106) = 1206.45, *p* < 0.001, η_p_^2^ = 0.84, and an interaction, *F*(45, 2106) = 38.87, *p* < 0.001, η_p_^2^ = 0.45, emerged. Expression effects were significant from the 301-to-400 ms interval onwards, ranging between *F*(5, 234) = 6.99, *p* < 0.001, η_p_^2^ = 0.13 and *F*(5, 234) = 70.02, *p* < 0.001, η_p_^2^ = 0.60. The post hoc multiple contrasts and the significant differences across expressions within each interval are shown in Fig. 2c. An advantage emerged for *happy* expressions over all the others, with the *threshold* located at the 401-to-500-ms interval: The smiling mouth region attracted more fixation time than the mouth region of all the other expressions, and the *amplitude* of this advantage remained until the end of the 1,000-ms display. Secondary advantages appeared for surprised and fearful faces, relative to sad and angry faces (see Fig. 2c).

### Potentially spurious results involving the nose/cheek region

The eye and the mouth regions are typically the most expressive sources in a face and, in fact, most of the statistical effects reported above emerged for these regions. Yet for disgusted (and, to a lesser extent, happy) expressions effects appeared also in the nose and cheek region (e.g., longer gaze duration). As indicated in the following analyses, these effects—rather than being spurious or irrelevant—can be explained as a function of morphological changes in the nose/cheek region of such expressions.

According to FACS (Facial Action Coding System) proposals^49^, facial *disgust* is typically characterized by AU9 (Action Unit; nose wrinkling or furrowing), which directly engages the nose/cheek region; and *happiness* is characterized by AU6 (cheek raiser) and AU12 (lip corner puller), which engage the mouth region and extend to the nose/cheek region. We used automated facial expression analysis^50,51^ by means of Emotient FACET SDK v6.1 software (iMotions; http://emotient.com/index.php) to assess these AUs in our stimuli. A one-way (6: Expression) ANOVA revealed higher AU9 scores for *disgusted* faces (*M* = 3.48) relative to all the others (ranging from −5.22 [surprise] to 0.19 [anger]), *F*(5, 234) = 134.46, *p* < 0.001, η_p_^2^ = 0.74. Relatedly, for *happy* faces, AU6 scores (*M* = 2.88) and AU12 (*M* = 4.06) scores were higher than for all the others, *F*(5, 234) = 126.00, *p* < 0.001, η_p_^2^ = 0.73 (AU6 ranging from to −2.32 [surprise] to 1.02 [disgust]), and *F*(5, 234) = 204.85, *p* < 0.001, η_p_^2^ = 0.81 (AU12 ranging from −1.80 [anger] to −0.76 [fear]), respectively.

## Discussion

The major goal of the present study was to investigate gaze behavior during recognition of dynamic facial expressions changing from neutral to emotional (happy, sad, angry, fearful, disgusted, or surprised). We determined selective attentional orienting to and engagement with expression-diagnostic regions; that is, those that have been found to contribute to (in that they are sufficient or necessary for) recognition^3–8^. As a secondary goal, we also aimed to validate a new stimulus set (KDEF-dyn) of dynamic facial expressions, and provide other researchers with norming data of categorization performance and eye fixation profiles for this instrument.

The relative recognition accuracies, efficiency, and confusions across expressions in the current study are consistent with those in prior research on emotional *expression categorization*. With *static* face stimuli, (a) recognition performance is typically higher for facial happiness, followed by surprise, which are higher than for sadness and anger, followed by disgust and fear^18,41,52,53^; (b) happy faces are recognized faster, and fear is recognized most slowly, across different response systems^4,53–55^; and (c) confusions occur mainly between disgust and anger, surprise and fear, and sadness and fear^28,38,55,56^. Regarding *dynamic* expressions in *on-line* video recordings, a pattern of recognition accuracies and reaction times comparable to ours (except for the lack of confusion of sadness as fear) has been found in prior research^19^. In addition, in studies using facial expressions in dynamic *morphing format*^18,38,41,57^, the pattern of expression recognition accuracy and confusions was also comparable to those in the current study. Thus, our recognition performance data concur with prior research data from static and dynamic expressions. This validates the KDEF-dyn set, and allows us to go forward and examine the central issues of the present approach concerning selective attention to dynamic expression-diagnostic face regions.

Our major contribution dealt with selective overt attention during facial expression processing, as reflected by eye movements and fixations. These measures have been obtained in many prior studies using static faces^3,15,58,59^, but scarcely in studies using dynamic faces^22,23^. Lischke *et al*.^22^ reported a trend towards longer gaze durations for expression-specific regions (i.e., the eyes of angry, sad, and fearful faces, and the mouth of happy faces). Our own results generally agree with these findings (except for fear) and extend them to additional expressions (disgust and surprise) and other eye-movement measures. In contrast, Blais *et al*.^23^ found no differences across the six basic expressions of emotion. However, as we argued in the Introduction, the lack of fixation differences in the Blais *et al*.^23^ study could be due to the use of a short stimulus display (500 ms) and a small stimulus size (5.72° width). In the current study (also in Lischke *et al*.^22^), we used longer displays (1,033 ms) and stimulus size (8.8° width) to increase sensitivity of measurement, which probably allowed for selective attention effects to emerge as a function of face region and expression.

**Figure 3:**
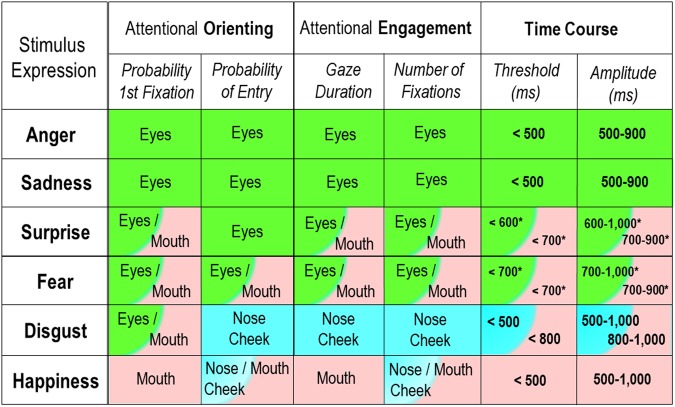
Summary of major findings of gaze behavior. Preferentially fixated (probability of first fixation, probability of entry, gaze duration, and number of fixations) face regions, and time course of fixation advantage (threshold, i.e., earliest time point; and amplitude, i.e., duration of advantage) across different expressions. Asterisks indicate a delayed and partial advantage for surprise and fear, relative to some—but not all—expressions (i.e., relative to disgust and happiness, for the eye region; or to sadness and anger, for the mouth region.

The current study addressed two aspects of selective visual attention to diagnostic features in dynamic expressions that were not considered previously: The distinction and time course of attentional orienting and engagement. As summarized in Fig. 3 (also Fig. 2a,b,c), the effects on orienting and engagement were generally convergent (except for minor discrepancies regarding disgusted faces): (a) *happy* faces were characterized by selective orienting to and engagement with the mouth region, which showed a time course advantage (i.e., both an earlier threshold and a longer amplitude of visual processing), relative to the other expressions; (b) *angry* and *sad* faces were characterized by orienting to and engagement with the eye region, with an earlier and longer time course advantage; (c) *disgusted* faces were characterized mainly by engagement with the nose/cheek, with a time course advantage; and (d) for *surprised* and *fearful* faces, both orienting and engagement were attracted by the eyes and the mouth in a balanced manner, with no dominance. This suggests that facial happiness, anger, sadness, and disgust processing relies on the analysis of single features (either the eyes or the mouth, or the nose), whereas facial surprise and fear processing would require a more holistic integration (see^60,61^). Further, our findings reveal a close relationship between expression-specific diagnostic regions^3–5,7,8^ and selective attention to them for dynamic (not only for static) facial expressions.

These findings have *theoretical implications* regarding the functional value of fixation profiles for expression categorization. It has been argued that fixation profiles reflect attention to the most diagnostic regions of a face for each emotion^8^. We have shown that the diagnostic facial features previously found to contribute to expression recognition^3–8^ are also the ones receiving earlier and longer overt attention during expression categorization. This allows us to infer that enhanced selective fixation on diagnostic regions of the respective expressions is functional for (i.e., facilitates) recognition. This is consistent with the hypothesis that observers move their eyes to face regions that maximize performance determining the emotional state of a face^26^, and the hypothesis of a predictive value of fixation patterns in recognizing emotional faces^14^. Nevertheless, beyond the aims and scope of the current study, an approach that directly addresses this issue should manipulate the visual availability or unavailability of diagnostic face regions, and examine how this affects actual expression recognition.

There are *practical implications* for an effective use of the current KDEF-dyn database: If the scanpath profiles when inspecting a face are functional (due to the diagnostic value of face regions), then such profiles can be taken as criteria for stimulus selection. We used a relatively large sample of stimuli (40 different models; 240 video-clips), which allows for selection of sub-samples depending on different research purposes (expression categorization, time course of attention, orienting, or engagement). Our stimuli vary in how much the respective scanpaths reflect the dominance (e.g., earlier first fixation, longer gaze duration, etc.) of diagnostic regions for each expression, and how much the scanpaths match the ideal pattern (e.g., earlier and longer gaze duration on the eye region of angry faces, etc.). This information can be obtained from our datasets (Supplemental Datasets S1A and S1B). Researchers could thus choose the stimulus models having the regions with enhanced attentional orienting or engagement, or a speeded time course (e.g., threshold) of attention. Of course, selection can also be made on the basis of recognition performance (hits, categorization efficiency, and type of confusions). Thus, the current study provides researchers with a useful methodological tool.

To conclude, we developed a set of morphed dynamic facial expressions of emotion (KDEF-dyn; see also^62^ for a complementary study using different measures). Expression recognition data were consistent with findings from prior research using static and other dynamic expressions. As a major contribution, eye-movement measures assessed selective attentional orienting and engagement, and its time course, for six basic emotions. Specific attentional profiles characterized each emotion: The eye region was looked at earlier and longer for angry and sad faces; the mouth region was looked at earlier and longer for happy faces; the nose/cheek region was looked at earlier and longer for disgusted faces; the eye and the mouth regions attracted attention in a more balanced manner for surprise and fear. This reveals selective visual attention to diagnostic features typically facilitating expression recognition.

## Electronic supplementary material

Supplementary Information

S1A Dataset

S1B Dataset

S1C Tables

## Data Availability

The authors declare that the data of the study are included in Supplemental Datasets S1A and S1B linked to this manuscript.

## References

[1] Ekman P, Cordaro D (2011). What is meant by calling emotions basic. Emotion Review.

[2] Calvo MG, Nummenmaa L (2016). Perceptual and affective mechanisms in facial expression recognition: An integrative review. Cogn Emot..

[3] Beaudry O, Roy-Charland A, Perron M, Cormier I, Tapp R (2014). Featural processing in recognition of emotional facial expressions. Cogn Emot..

[4] Calder AJ, Young AW, Keane J, Dean M (2000). Configural information in facial expression perception. Journal of Experimental Psychology Human Perception and Performance.

[5] Calvo MG, Fernández-Martín A, Nummenmaa L (2014). Facial expression recognition in peripheral versus central vision: Role of the eyes and the mouth. Psychological Research.

[6] Kohler CG (2004). Differences in facial expressions of four universal emotions. Psychiatry Res..

[7] Smith ML, Cottrell GW, Gosselin F, Schyns PG (2005). Transmitting and decoding facial expressions. Psychological Science.

[8] Schurgin MW (2014). Eye movements during emotion recognition in faces. Journal of Vision.

[9] Calvo MG, Nummenmaa L (2008). Detection of emotional faces: salient physical features guide effective visual search. J Exp Psychol Gen..

[10] Ebner NC, He Y, Johnson MK (2011). Age and emotion affect how we look at a face: visual scan patterns differ for own-age versus other-age emotional faces. Cogn Emot..

[11] Eisenbarth H, Alpers GW (2011). Happy mouth and sad eyes: Scanning emotional facial expressions. Emotion.

[12] Bombari D (2013). Emotion recognition: The role of featural and configural face information. Quarterly Journal of Experimental Psychology.

[13] Jack RE, Blais C, Scheepers C, Schyns PG, Caldara R (2009). Cultural confusions show that facial expressions are not universal. Curr Biol..

[14] Vaidya AR, Jin C, Fellows LK (2014). Eye spy: The predictive value of fixation patterns in detecting subtle and extreme emotions from faces. Cognition.

[15] Wells LJ, Gillespie SM, Rotshtein P (2016). Identification of emotional facial expressions: effects of expression, intensity, and sex on eye gaze. PloS ONE.

[16] Wong B, Cronin-Golomb A, Neargarder S (2005). Patterns of visual scanning as predictors of emotion identification in normal aging. Neuropsychology.

[17] Krumhuber EG, Kappas A, Manstead ASR (2013). Effects of dynamic aspects of facial expressions: A review. Emotion Review.

[18] Calvo MG, Avero P, Fernandez-Martin A, Recio G (2016). Recognition thresholds for static and dynamic emotional faces. Emotion.

[19] Wingenbach TS, Ashwin C, Brosnan M (2016). Validation of the Amsterdam Dynamic Facial Expression Set - Bath Intensity Variations (ADFES-BIV): A set of videos expressing low, intermediate, and high intensity emotions. PloS ONE.

[20] Arsalidou M, Morris D, Taylor MJ (2011). Converging evidence for the advantage of dynamic facial expressions. Brain Topography.

[21] Trautmann SA, Fehr T, Herrmann M (2009). Emotions in motion: Dynamic compared to static facial expressions of disgust and happiness reveal more widespread emotion-specific activations. Brain Research.

[22] Lischke A (2012). Intranasal oxytocin enhances emotion recognition from dynamic facial expressions and leaves eye-gaze unaffected. Psychoneuroendocrinology.

[23] Blais C, Fiset D, Roy C, Saumure-Régimbald C, Gosselin F (2017). Eye fixation patterns for categorizing static and dynamic facial expressions. Emotion.

[24] Hoffmann H, Traue HC, Bachmayr F, Kessler H (2010). Perceived realism of dynamic facial expressions of emotion: Optimal durations for the presentation of emotional onsets and offsets. Cogn Emot..

[25] Krumhuber EG, Skora L, Küster D, Fou L (2017). A review of dynamic datasets for facial expression research. Emotion Review.

[26] Peterson MF, Eckstein MP (2012). Looking just below the eyes is optimal across face recognition tasks. PNAS.

[27] Lundqvist, D., Flykt, A. & Öhman, A. The Karolinska Directed Emotional Faces–KDEF [CD-ROM]. *Department of Clinical Neuroscience, Psychology section, Karolinska Institutet, Stockholm, Sweden* ISBN 91-630-7164-9 (1998).

[28] Calvo MG, Lundqvist D (2008). Facial expressions of emotion (KDEF): Identification under different display-duration conditions. Behavior Research Methods.

[29] Goeleven E, De Raedt R, Leyman L, Verschuere B (2008). The Karolinska Directed Emotional Faces: A validation study. Cogn Emot..

[30] Calvo MG, Gutiérrez-García A, Avero P, Lundqvist D (2013). Attentional mechanisms in judging genuine and fake smiles: Eye-movement patterns. Emotion.

[31] Gupta R, Hur YJ, Lavie N (2016). Distracted by pleasure: Effects of positive versus negative valence on emotional capture under load. Emotion.

[32] Sanchez A, Vazquez C, Gómez D, Joormann J (2014). Gaze-fixation to happy faces predicts mood repair after a negative mood induction. Emotion.

[33] Adamaszek M (2015). Neural correlates of impaired emotional face recognition in cerebellar lesions. Brain Research.

[34] Bublatzky F, Gerdes AB, White AJ, Riemer M, Alpers GW (2014). Social and emotional relevance in face processing: Happy faces of future interaction partners enhance the late positive potential. Frontiers in Human Neuroscience.

[35] Calvo MG, Beltrán D (2014). Brain lateralization of holistic versus analytic processing of emotional facial expressions. NeuroImage.

[36] Pollick FE, Hill H, Calder A, Paterson H (2003). Recognising facial expression from spatially and temporally modified movements. Perception.

[37] Fiorentini C, Viviani P (2011). Is there a dynamic advantage for facial expressions?. Journal of Vision.

[38] Recio G, Schacht A, Sommer W (2013). Classification of dynamic facial expressions of emotion presented briefly. Cogn Emot..

[39] Harris RJ, Young AW, Andrews TJ (2014). Dynamic stimuli demonstrate a categorical representation of facial expression in the amygdala. Neuropsychologia.

[40] Popov T, Miller GA, Rockstroh B, Weisz N (2013). Modulation of alpha power and functional connectivity during facial affect recognition. The Journal of Neuroscience: The official journal of the Society for Neuroscience.

[41] Recio G, Schacht A, Sommer W (2014). Recognizing dynamic facial expressions of emotion: Specificity and intensity effects in event-related brain potentials. Biological Psychology.

[42] Vrticka P, Lordier L, Bediou B, Sander D (2014). Human amygdala response to dynamic facial expressions of positive and negative surprise. Emotion.

[43] Hess U, Kappas A, McHugo GJ, Kleck RE, Lanzetta JT (1989). An analysis of the encoding and decoding of spontaneous and posed smiles: The use of facial electromyography. Journal of Nonverbal Behavior.

[44] Weiss F, Blum GS, Gleberman L (1987). Anatomically based measurement of facial expressions in simulated versus hypnotically induced affect. Motivation & Emotion.

[45] Faul F, Erdfelder E, Lang AG, Buchner A (2007). G*Power 3: A flexible statistical power analysis program for the social, behavioral, and biomedical sciences. Behavior Research Methods.

[46] Schultz J, Pilz KS (2009). Natural facial motion enhances cortical responses to faces. Experimental Brain Research.

[47] Johnston P, Mayes A, Hughes M, Young AW (2013). Brain networks subserving the evaluation of static and dynamic facial expressions. Cortex.

[48] Holmqvist, K., Nyström, N., Andersson, R., Dewhurst, R., Jarodzka, H., & Van de Weijer, J. *Eye tracking: A comprehensive guide to methods and measures* (Oxford University Press, Oxford, UK, 2011).

[49] Ekman, P., Friesen, W. V. & Hager, J. C. *Facial action coding system* (A Human Face, Salt Lake City, 2002).

[50] Cohn, J. F. & De la Torre, F. Automated face analysis for affective computing. In: Calvo, R. A., Di Mello, S., Gratch, J. & Kappas, A. (editors). *The Oxford handbook of affective computing*, 131–151 (Oxford University Press, New York, 2015).

[51] Bartlett, M. & Whitehill, J. Automated facial expression measurement: Recent applications to basic research in human behavior, learning, and education. In: Calder, A., Rhodes, G., Johnson, M. & Haxby, J. (editors). *Handbook of face perception*, 489–513 (Oxford University Press, Oxford, UK, 2011).

[52] Nelson NL, Russell JA (2013). Universality revisited. Emotion Review.

[53] Calvo MG, Nummenmaa L (2009). Eye-movement assessment of the time course in facial expression recognition: Neurophysiological implications. Cognitive, Affective & Behavioral Neuroscience.

[54] Elfenbein HA, Ambady N (2003). When familiarity breeds accuracy: Cultural exposure and facial emotion recognition. Journal of Personality and Social Psychology.

[55] Palermo R, Coltheart M (2004). Photographs of facial expression: Accuracy, response times, and ratings of intensity. Behavior Research Methods, Instruments, & Computers.

[56] Tottenham N (2009). The NimStim set of facial expressions: Judgments from untrained research participants. Psychiatry Research.

[57] Langner O (2010). Presentation and validation of the Radboud Faces Database. Cogn Emot..

[58] Hsiao JH, Cottrell G (2008). Two fixations suffice in face recognition. Psychological Science.

[59] Kanan C, Bseiso DN, Ray NA, Hsiao JH, Cottrell GW (2015). Humans have idiosyncratic and task-specific scanpaths for judging faces. Vision Research.

[60] Meaux E, Vuilleumier P (2016). Facing mixed emotions: Analytic and holistic perception of facial emotion expressions engages separate brain networks. NeuroImage.

[61] Tanaka JW, Kaiser MD, Butler S, Le Grand R (2012). Mixed emotions: Holistic and analytic perception of facial expressions. Cogn Emot..

[62] Calvo, M. G., Fernández-Martín, A., Recio, G. & Lundqvist, D. Human observers and automated assessment of dynamic emotional facial expressions: KDEF-dyn database validation. *Frontiers in Psychology* **9**:2052 (2018). 10.3389/fpsyg.2018.02052 PMC6212581 30416473

