# Supplementary material for: Selective eye fixations on diagnostic face regions of dynamic emotional expressions: KDEF-dyn database
Source: Sci Rep. 2018 Nov 19;8:17039. doi: 10.1038/s41598-018-35259-w (PMC6242984; doi:10.1038/s41598-018-35259-w)
Supplement: Supplementary file 1 — Supplementary Information [file 41598_2018_35259_MOESM1_ESM.pdf]

Selective eye fixations on diagnostic face regions

of dynamic emotional expressions: KDEF-dyn database

Manuel G. Calvo*, Andrés Fernández-Martín, Aida Gutiérrez-García,

and Daniel Lundqvist

*mgcalvo@ull.edu.es

Obtaining the database. The KDEF-dyn stimuli and supplemental materials are available for scientific purposes, and can be downloaded from http://kdef.se/versions.html (KDEF-dyn II).

S2 Stimuli. Video-clip stimuli_MP4. 240 video-clips, separated for each of six emotional expression categories (40 video-clips each). Available at http://kdef.se/versions.html

S3 Graphs. Time-course graphs. In the graphical representation of *time course*, only *net gaze* duration for each region (eyes, nose, and mouth) is shown. This reflects ~85% of total (100%) fixation time, (1) after saccades, blinks, and fixations < 80 ms were excluded (see *Design and measures* section), and (2) after fixations outside target regions were removed. The remaining ~15% of fixation time corresponds to (1) and (2). Fixation time in the time-course graphs was averaged for consecutive periods of 20 ms. Available at http://kdef.se/versions.html

S4 Heatmaps. Video-clips of visual-scan heatmaps. For *heatmaps*, all fixations and regions are included. The dynamic heatmaps are played in slow motion, i.e., the original 1,033-ms video-clip display is shown for 4 s. Available at http://kdef.se/versions.html
