# Supplementary material for: Selective eye fixations on diagnostic face regions of dynamic emotional expressions: KDEF-dyn database
Source: Sci Rep. 2018 Nov 19;8:17039. doi: 10.1038/s41598-018-35259-w (PMC6242984; doi:10.1038/s41598-018-35259-w)
Supplement: Supplementary file 4 — S1C Tables [file 41598_2018_35259_MOESM4_ESM.pdf]

Selective eye fixations on diagnostic face regions of dynamic emotional expressions: KDEF-dyn database

Manuel G. Calvo*, Andrés Fernández-Martín, Aida Gutiérrez-García, and Daniel Lundqvist

*mgcalvo@ull.edu.es

S1C Tables. Table 6a (linked with Figure 2a in the article). Time Course of Fixation Duration on Face Regions. Mean scores (*M*) and standard errors (*SE*) of proportion of fixation time on the EYE REGION across intervals.

EXPRESSION	Interval																			
	1-100 ms		101-200 ms		201-300 ms		301-400 ms		401-500 ms		501-600 ms		601-700 ms		701-800 ms		801-900 ms		901-1000 ms	
	M	*SE*	*M*	*SE*	*M*	*SE*	*M*	*SE*	*M*	*SE*	*M*	*SE*	*M*	*SE*	*M*	*SE*	*M*	*SE*	*M*	*SE*
**ANGER**	25.11	0.59	25.70	0.59	34.94	0.64	41.17	0.74	44.85	0.88	46.66	1.07	47.97	1.18	49.67	1.18	49.95	1.17	49.85	1.16
**DISGUST**	25.61	0.59	26.29	0.59	32.95	0.64	38.26	0.74	39.35	0.88	39.02	1.07	37.37	1.18	37.21	1.18	39.22	1.17	40.28	1.16
**FEAR**	24.06	0.59	25.73	0.59	34.91	0.64	40	0.74	41.10	0.88	41.66	1.07	42.47	1.18	43.44	1.18	44.17	1.17	45.85	1.16
**HAPPINESS**	24.52	0.59	25.67	0.59	32.82	0.64	36.70	0.74	34.74	0.88	29.94	1.07	24.82	1.18	23.03	1.18	24.44	1.17	27.02	1.16
**SADNESS**	25.24	0.59	25.81	0.59	35.54	0.64	41.98	0.74	45.01	0.88	46.48	1.07	47.94	1.18	49.80	1.18	49.92	1.17	48.96	1.16
**SURPRISE**	25.42	0.59	26.59	0.59	33.92	0.64	39.07	0.74	40.87	0.88	41.80	1.07	42.74	1.18	44.45	1.18	44.90	1.17	46.39	1.16

S1C Tables. Table 6b (linked with Figure 2b in the article). Time Course of Fixation Duration on Face Regions. Mean scores (*M*) and standard errors (*SE*) of proportion of fixation time on the NOSE REGION across intervals.

EXPRESSION	Interval																			
	1-100 ms		101-200 ms		201-300 ms		301-400 ms		401-500 ms		501-600 ms		601-700 ms		701-800 ms		801-900 ms		901-1000 ms	
	M	*SE*	*M*	*SE*	*M*	*SE*	*M*	*SE*	*M*	*SE*	*M*	*SE*	*M*	*SE*	*M*	*SE*	*M*	*SE*	*M*	*SE*
**ANGER**	58.03	0.67	58.25	0.66	43.95	0.75	33.01	0.75	29.25	0.75	27.32	0.80	25.39	0.80	24.37	0.81	23.95	0.80	24.33	0.83
**DISGUST**	57.67	0.67	57.94	0.66	45.94	0.75	35.80	0.75	33.54	0.75	32.94	0.80	32.41	0.80	31.57	0.81	30.56	0.80	30.10	0.83
**FEAR**	58.54	0.67	58.21	0.66	43.97	0.75	33.63	0.75	30.20	0.75	28.45	0.80	27.33	0.80	26.58	0.81	25.90	0.80	25.36	0.83
**HAPPINESS**	59.19	0.67	58.74	0.66	46.17	0.75	35.18	0.75	31.02	0.75	30.54	0.80	30.17	0.80	29.44	0.81	27.83	0.80	26.30	0.83
**SADNESS**	57.99	0.67	58.01	0.66	44.30	0.75	33.38	0.75	29.12	0.75	27.30	0.80	25.37	0.80	24.20	0.81	24.53	0.80	24.89	0.83
**SURPRISE**	58.40	0.67	57.85	0.66	45.12	0.75	33.84	0.75	28.77	0.75	26.72	0.80	25.55	0.80	23.91	0.81	23.57	0.80	23.03	0.83

S1C Tables. Table 6c (linked with Figure 2c in the article). Time Course of Fixation Duration on Face Regions. Mean scores (*M*) and standard errors (*SE*) of proportion of fixation time on the MOUTH REGION across intervals.

EXPRESSION	Interval																			
	1-100 ms		101-200 ms		201-300 ms		301-400 ms		401-500 ms		501-600 ms		601-700 ms		701-800 ms		801-900 ms		901-1000 ms	
	M	*SE*	*M*	*SE*	*M*	*SE*	*M*	*SE*	*M*	*SE*	*M*	*SE*	*M*	*SE*	*M*	*SE*	*M*	*SE*	*M*	*SE*
**ANGER**	2.13	0.27	2.19	0.29	7.47	0.42	13.30	0.63	15.29	0.83	15.42	0.89	14.96	0.97	14.37	0.98	14.41	0.91	13.71	0.84
**DISGUST**	1.84	0.27	2.13	0.29	7.37	0.42	13.73	0.63	16.64	0.83	17.48	0.89	19.21	0.97	19.78	0.98	19.29	0.91	18.54	0.84
**FEAR**	2.64	0.27	2.85	0.29	8.20	0.42	14.46	0.63	16.98	0.83	18.80	0.89	20.20	0.97	19.90	0.98	18.84	0.91	18.41	0.84
**HAPPINESS**	2.25	0.27	2.35	0.29	8.20	0.42	17.46	0.63	23.26	0.83	28.97	0.89	34.07	0.97	36.45	0.98	35.03	0.91	32.08	0.84
**SADNESS**	2.17	0.27	2.41	0.29	7.19	0.42	13.13	0.63	14.96	0.83	15.76	0.89	15.56	0.97	14.76	0.98	14.68	0.91	14.37	0.84
**SURPRISE**	2.13	0.27	2.25	0.29	8.01	0.42	15.56	0.63	19.23	0.83	20.30	0.89	20.54	0.97	20.61	0.98	19.32	0.91	18.08	0.84
